# Carcinoma of the Stomach

**Published:** 1891-03

**Authors:** A. Garwood

**Affiliations:** New Braunfels


For Daniel's Texas Medical Journal.

### CARCINOMA OF THE STOMACH.—A CASE.

BY A. GARWOOD, M. D., NEW BRAUNFELS.

Read before West Texas Medical Association.

CANCEROUS diseases of whatever form, are always especially interesting to the physician and surgeon, and are perhaps dreaded by the laity above all other afflictions.

But, notwithstanding the importance of the subject, and the great advance in medical and sanitary science, the fact remains that cancerous afflictions are increasing among us.

I will not trouble you with figures or statistics as prepared by Sir James Paget, Dr. Fordyce Barker and others, in proof of this assertion. This fact, however, coupled with the knowledge that the disease lays hold of its victims at the most active and best period of life, gives greatest importance to the subject.

I invite your attention to carcinoma of the stomach, because the disease is oftener found in this organ than in any other part of the alimentary canal, excepting the tongue and lips, and yet it is commonly overlooked in its incipiency, and is not easy of diagnosis even when well advanced.

There is probably not one among us, who has not experienced the mortification of mistaking this most formidable disease for a trivial affection, until our confiding patient was beyond redemption, if any there be for such an affliction.

The usual varieties of carcinoma affecting the stomach are scirrhus, encephaloid and colloid—the most common form being scirrhus and is usually found at the pylorus. To discuss these different forms, their pathology, or ætiology would take me beyond the intended limits of this paper, and besides, I may as well confess, I have not the ability to do so. From books one may read as well as another, and to these I refer you. It is certain that cancer is prone to develop from an irritated surface or from scar-tissue which, possibly, explains why the pyloric end of the stomach is oftenest affected, and is an argument, too, in favor of a local origin of the disease. It is easy to understand, how the mucous membrane of the pylorus, might be abraded and irritated by the passage of hard or indigestible masses of food, and it is a matter of fact, that old cicatrices are often found post mortem in this region.

The affections simulating cancer of the stomach are: Gastrodynia, atonic dyspepsia, gastric catarrh, fibrous stenosis of pylorus and gastric ulcer.

All are familiar with the ordinary diagnostic indications: The age and sex of the patient, character of pain, locality, vomiting, hæmatemesis, tumor felt on palpation, the subjective complaints of the patient, etc.

Within the last few years, great helps to diagnosis have been furnished by urine and blood analysis, but the most satisfactory aid comes from a study of the chemical conditions of the stomach itself.

Rommelaers was first to call attention to the diminution of urea and phosphates in cancer. He asserts that when the daily amount of urea falls below twelve grammes, we should suspect cancer. Maixnar found peptone uniformily present in gastric cancer, but does not state, whether or not, it is equally frequent in gastric ulcer.

M. Hagen, in his work on the pathology of the blood, says that in cancer the red corpuscles may become very much reduced in numbers, and structural alterations occur, which simulate the appearance of parasites, and that in cancer of the stomach the hæmoglobin is diminished fifty per cent.

In 1871 Leube advocated the use of the stomach tube for diagnostic purposes in stomach diseases. Within the last decade, numerous workers have been attracted to this new field, and the result is, that we now have a voluminous literature on the subject. Improved methods and apparatus are now at our command, for surely and quickly diagnosing stomach diseases, and this, too, without much discomfort to the patient, or great loss of time to ourselves.

An ingenious method of illuminating the stomach from within, has been devised by Dr. Einhorn, of New York. It consists of an Edison's incandescent lamp inserted into the tip of an ordinary soft rubber stomach tube, the conductor being carried through the tube, and attached to a storage battery. In a lean or cancerous subject the contour of the stomach is at once seen through the abdominal walls, and the size of the stomach is plainly demonstrated. The presence of a tumor located anteriorly may be surmised by an increased dimness of the stomach outline at that point.

If there is cancer of the pylorus, the stomach is distended, if of the cardiac orifice, the stomach is contracted. In carcinoma of the stomach absorption of contents is greatly delayed.

Recently I have employed Dr. Einhorn's stomach bucket in diagnosing stomach troubles, and I take pleasure in recommending it to those of the profession not acquainted with its use.

It is the latest, and I believe, best device for obtaining the stomach contents for diagnostic purposes. As you may see, it is a very simple little instrument. But little larger than a o o capsule, (which it resembles in shape), it is scarcely harder to swallow.

It is made of silver, and at its top there is a large opening with an arch over it. On this arch, a silk thread about 12 inches in length, is tied, for the purpose of withdrawing the bucket when filled.

As to method of using, and chemical tests, I will read from the printed directions, accompanying each apparatus. * * * It is at once evident that in all stomach troubles, the determination of free h. c. e. plays a main role, for as all know, it forms one of the most important products of stomach secretion.

The absence of h. c. e. in cancer of the stomach, was first discovered by Von den Velden, but it has since been demonstrated that there may be a diminution, or absence of this acid in chronic gastritis and glandular atrophy of the mucous membrane—so, while this certainly detracts from its *positive* value, it still remains a most important factor where there exists other symptoms and signs of the disease. If, however, h. c. e. is repeatedly found to be present after examinations in doubtful cases, we are *positively certain* of the *absence* of cancer, no matter how strong the signs and symptoms may have pointed to that disease. The rennet ferment is also usually absent in cancerous disease.

As to treatment of cancer of the stomach, I have little to say. I do not know, personally, of a single recovery, though I have read within the past year of two cures by the knife. I believe it most practical to regard the disease as a local one, which, if given into the hands of the surgeon at an early stage, may be completely eradicated.

